# High levels of serum cholesterol increase the risk of developing vessel co‐opting tumors in colorectal cancer liver metastases

**DOI:** 10.1111/febs.70348

**Published:** 2025-11-28

**Authors:** Amatzia Gantz, Audrey Kapelanski‐Lamoureux, Miran Rada, Lucyna Krzywon, Migmar Tsamchoe, Anastasia Tsatoumas, Stephanie K. Petrillo, Nabil G. Seidah, Anthoula Lazaris, Peter Metrakos

**Affiliations:** ^1^ Department Anatomy and Cell Biology McGill University Quebec Canada; ^2^ Cancer Research Program Research Institute of the McGill University Health Centre Montreal Canada; ^3^ Laboratory of Biochemical Neuroendocrinology Clinical Research Institute of Montreal Canada

**Keywords:** angiogenesis, cholesterol, PCSK9, sex stratification, vessel co‐option

## Abstract

Cholesterol availability has been implicated in cancer progression. Here, we demonstrate the connection between blood cholesterol concentration, the development of distinct histopathological growth patterns (HGPs) in colorectal cancer liver metastases (CRCLM), and new avenues of treatment for patients. Our study reveals that serum cholesterol levels correlate with the different HGPs, and patients on statins had desmoplastic HGPs and improved overall survival. We also demonstrate, in an *in vivo* mouse model, that inhibition of key modulators of cholesterol metabolism, using antiproprotein convertase subtilisin/kexin type 9 (PCSK9) inhibitors, attenuated the development of CRCLM tumors, as well as the formation of vessel co‐opting lesions. Our findings underscore the importance of cholesterol in CRCLM and the potential clinical application of evolocumab to mitigate the growth of these histological types of tumors in CRCLM.

## Graphical abstract

High serum cholesterol is linked to colorectal cancer liver metastases (CRCLM) subtypes with poor survival (replacement pattern). Levels of cholesterol under 4 mmol·L^−1^ correlated with better survival and a desmoplastic pattern subtype. Furthermore, *in vivo* PCSK9 inhibitor evolocumab, which lowers cholesterol, reduced liver metastases. These findings highlight cholesterol's role in CRCLM and the therapeutic potential of evolocumab in slowing tumor growth in affected patients.

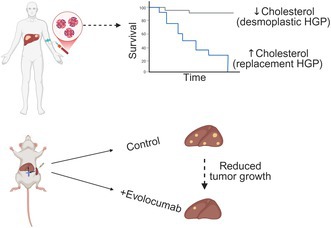

**Abbreviations**
CRC: colorectal cancer
CRCLM: colorectal cancer liver metastases
DHGP: desmoplastic histopathological growth pattern
EDNO: endothelium‐ derived nitric oxide
FFPE: formalin‐fixed and paraffin‐embedded
HGP: histopathological growth pattern
HGPs: histopathological growth patterns
HMGCR: HMG‐CoA reductase (3‐hydroxy‐3‐methylglutaryl‐coenzyme A reductase)
LDL: low‐density lipoprotein
LDL‐C: low‐density lipoprotein cholesterol
LM: liver metastases
PCSK9: proprotein convertase subtilisin/kexin type 9
RHGP: replacement histopathological growth pattern/vessel co‐option
ROI: regions of interest

## Introduction

Numerous reports suggest a role for cholesterol availability in the progression of cancer. Given the epidemiological and preclinical data supporting a protective role of statins, ezetimibe and cholesterol disruption in multiple cancers (prostate, HCC, ovarian, breast, etc.) [1, 2], clinical trials assessing the feasibility, safety, and efficacy of a multipathway cholesterol depletion in addition to standard therapy are on the rise. Various studies indicated an association between the risk of developing cancer and the elevation of serum cholesterol levels [3]. Cholesterol, a vital lipid molecule, plays a crucial role in maintaining cellular homeostasis throughout the body, and the liver is the main organ for its biosynthesis [4, 5, 6].

The discovery of Proprotein Convertase Subtilisin/Kexin Type 9 (PCSK9) in 2003 [7] and its implication in the regulation of low‐density lipoprotein cholesterol (LDL‐C) [8] was a game changer in the development of more efficient treatments for hypercholesterolemia and associated cardiovascular diseases [9, 10]. Emerging evidence has shown that PCSK9 contributes to various aspects of cancer, such as cell proliferation, apoptosis, invasion, metastasis, antitumor immunity, and radioresistance, strengthening the hypothesis that PCSK9 could be a promising therapeutic target [11, 12, 13, 14, 15]. In this context, a study conducted by Liu *et al*. [16] demonstrated that inhibition of PCSK9 using PCSK9‐neutralizing antibodies led to a reduction in tumor growth from various cancer cells in mouse models. This antitumor effect was found to be dependent on the presence of CD45+ leukocytes, and more specifically, CD8+ T cells, suggesting a potential immunomodulatory mechanism for PCSK9 inhibition in cancer therapy. Moreover, PCSK9 inhibition potentiated the efficacy of anti‐PD1 immunotherapy in mice [16]. On the other hand, Xu *et al*. [17] have demonstrated that PCSK9 promotes gastric cancer metastasis and suppresses apoptosis through HSP70 upregulation. PCSK9 might also be involved in metabolism reprogramming in tumors through the regulation of the receptors and transporters that are potentially involved in lipid metabolism regulation, such as LDLR, LRP1, VLDLR, APOER2, CD36, ABCA1, and NPC1L1 [18, 19]. In this context, PCSK9 has been reported to impair apoptosis in hepatocellular carcinoma cells via upregulation of FASN expression [20]. However, the role of PCSK9 in CRCLM is still largely unknown.

Recent research has revealed a promising role for PCSK9 inhibition in the treatment of liver cancer, particularly hepatocellular carcinoma (HCC). Targeting PCSK9 alone has been shown to significantly reduce tumor cell proliferation and migration, indicating a potential antitumor effect through suppression of key cancer‐promoting pathways [20]. Moreover, silencing of PCSK9 was associated with the induction of ferroptosis—an iron‐dependent form of regulated cell death characterized by lipid peroxidation [21]. Mechanistically, PCSK9 depletion leads to a downregulation of the antioxidant regulator Nrf2, compromising the cellular defense system against oxidative stress and thereby sensitizing cells to ferroptotic death [21]. These findings suggest that PCSK9 blockade, whether via monoclonal antibodies, small‐molecule inhibitors, or siRNA, may offer a novel therapeutic strategy, either as monotherapy or in combination with agents such as statins [21, 22]. It is important to note, however, that these studies have been conducted primarily in models of hepatocellular carcinoma, a primary liver malignancy often arising from viral hepatitis or metabolic liver disease. Unlike HCC, which arises from chronic hepatic inflammation or metabolic dysfunction, CRCLM originates from a primary tumor in the colon and adapts to the liver microenvironment during metastatic colonization. This key difference suggests that the metabolic dependencies and immunomodulatory roles of PCSK9 may differ significantly between these two cancer types. Nevertheless, several hypotheses warrant exploration. PCSK9 is known to mediate degradation of LDL receptors and other immune regulatory surface proteins such as MHC‐I. In the context of metastasis, this could influence both tumor lipid metabolism and immune evasion. Furthermore, liver metastases from colorectal cancer often develop in lipid‐ and inflammation‐rich environments, where PCSK9 expression and function may contribute to metastatic niche formation or resistance to therapy. Given these uncertainties, extrapolation from HCC must be done cautiously. While the ferroptosis‐sensitizing and immunomodulatory effects of PCSK9 depletion observed in HCC are compelling, the distinct biological context of CRCLM, particularly its clonal origin and interaction with the hepatic immune landscape, requires dedicated investigation.

Monoclonal antibody PCSK9 (evolocumab) and HMG‐CoA reductase (HMGCR, statins) inhibitors play crucial roles in regulating cholesterol metabolism and tissue uptake [23]. Ezetimibe inhibits cholesterol absorption by the gut, thus reducing overall delivery of cholesterol to the liver, promoting synthesis of LDL‐R with subsequent reduction in serum LDL‐C. In preclinical mouse models, it has been shown that dietary cholesterol increases the incidence and severity of liver cancer [24, 25]. Cancer cells have been shown to require high levels of cholesterol to sustain tumor growth [26]. Accordingly, it has also been shown that inhibition of PCSK9 may starve cancer cells [26] and PCSK9 inhibition also substantially reduces melanoma metastasis to the liver [27] and lung [28] in mice. Therefore, inhibiting PCSK9 could potentially assault the tumor by depriving it of cholesterol and thus potentiating cancer cell targeting by combinational strategies.

Colorectal cancer (CRC) is the third most common cancer and the second leading cause of cancer‐related deaths worldwide [29, 30]. The liver is the most common site of metastases of CRC, with approximately 50% of patients diagnosed with CRC developing liver metastasis (LM) during the course of the disease impacting prognosis and treatment strategies for CRC patients [31, 32]. Surgical resection is recognized as the gold standard treatment, improving survival by up to 50% and offering CRCLM patients a potentially curative treatment [33]. However, only 10–20% of CRCLM patients are eligible for surgical resection [34]. Among the remaining patients, while a combination of antiangiogenic agents (e.g., Bevacizumab: Bev) with chemotherapy (chemo) showed improvement in survival among CRCLM patients, recurrence is still common [35].

We and others have identified two main CRCLM histologic growth patterns (HGPs) that predict response to treatment/survival: (1) the desmoplastic HGP (DHGP), characterized by a desmoplastic stroma separating CRC cancer cells from the liver parenchyma; and (2) the replacement HGP (RHGP) where tumor cells infiltrate the parenchymal cells in the liver as the lesions expand [36]. Furthermore, DHGP lesions show ongoing angiogenesis and are characteristically associated with an intense inflammatory infiltrate at the tumor–stromal interface [36, 37]. In contrast, RHGP lesions grow by co‐opting the sinusoidal blood vessels between the liver cell plates without sprouting angiogenesis, with little perturbation of the liver architecture [36, 38, 39]. Treatment given to resected CRCLM patients with predominantly DHGP metastasis receiving neoadjuvant Bev + chemo has more than doubled the 5‐year overall survival compared to patients with RHGP who have received the same neoadjuvant regimen [36]. As of now, there is no effective treatment for patients with RHGP lesions.

We present data demonstrating upregulation of PCSK9 in CRCLM lesions, and its inhibition attenuating tumor growth and RHGP lesions (co‐opting vessel formation) in a mouse metastatic preclinical model. Moreover, blocking the LDLR‐degradation activity by PCSK9 *in vivo* using a monoclonal antibody, evolocumab, elevated the intratumoral levels of CD8^+^ T cells, while dramatically reducing the intratumoral macrophages. Interestingly, the intratumoral macrophages impede CD8^+^ T cells from reaching tumor cells and limit the efficacy of immunotherapy [40]. Furthermore, we demonstrate that high cholesterol levels in patients' blood correlate with the presence of RHGP lesions. Taken together, our data suggest the importance of cholesterol in the development of RHGP lesions and since PCSK9‐neutralizing antibodies including evolocumab and alirocumab are clinically approved to treat hyperlipidemia in humans [41], inhibiting PCSK9 through these antibodies may represent an effective strategy for anticancer therapy in CRCLM.

## Results

### Preoperative serum total cholesterol and LDL score as a novel predictor of the type of HGPs and overall survival in CRCLM patients

To investigate the association between serum cholesterol levels in CRCLM patients and the presence of RHGP lesions (vessel co‐option tumors), we analyzed the total cholesterol and LDL in 87 CRCLM patients, of whom 64.4% were male and 35.6% were female. Based on the 50% cutoff predominant HGPs scoring of the tumors, the patients were divided into three groups: vessel co‐opting RHGP (*n* = 42: 39.2%), angiogenic DHGP (*n* = 40: 37.4%), and mixed lesions with a pushing phenotype (*n* = 25: 23.4%). For this study, we included all patient samples for the cholesterol analysis when looking at male and female differences and focused only on the two main HGPs (RHGP and DHGP) for the rest of the analyses.

**Fig. 1:**
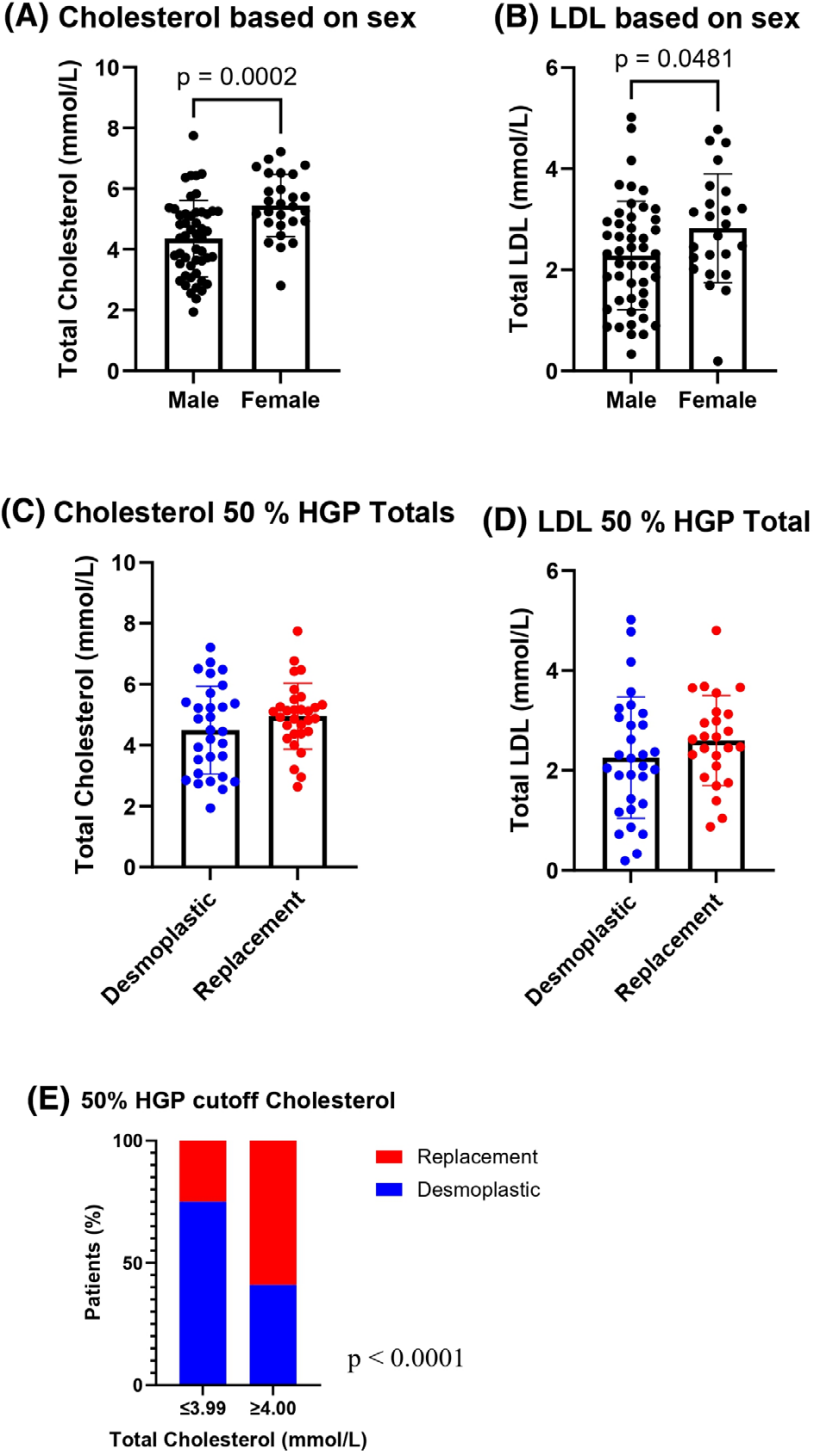
Serum Cholesterol and LDL levels in colorectal cancer liver metastasis (CRCLM) patients stratified by sex and HGP. (A) Total cholesterol levels based on sex stratification. Unpaired *t*‐test, *P* = 0.0002. (male *n* = 50, female *n* = 27). (B) Low‐density lipoprotein (LDL) levels based on sex stratification. Unpaired *t*‐test, *P* = 0.0481 (male *n* = 48, female *n* = 24). (C) Cholesterol levels based on HGP stratification (*n* = 60). (D) LDL levels based on histological growth pattern (HGP) stratification (*n* = 55). (E) Patients percentage stratification by 50% HGP cutoff, divided based on the concentration of total serum cholesterol. Chi‐square test, *P* < 0.0001 (*n* = 60). Error bars denote standard deviation.

As shown in Fig. 1A, the total serum cholesterol was significantly higher in female patients than in male patients. A significant difference in serum LDL levels was also observed between male and female patients (Fig. 1B). Comparing the RHGP and DHGP groups without sex stratification resulted in no significant difference whether looking at cholesterol or LDL (Fig. 1C,D). When stratifying patients by cholesterol levels, we noticed a significant difference in HGPs. Patients with lower than 4 mmol·L^−1^ total cholesterol tend to have more desmoplastic lesions, whereas patients with higher cholesterol (≥ 4.00 mmol·L^−1^) presented a significant increase in replacement lesions (*P* < 0.0001, Fig. 1E).

### Expression profiles of lipid metabolism differ between replacement and desmoplastic growth patterns

**Fig. 2:**
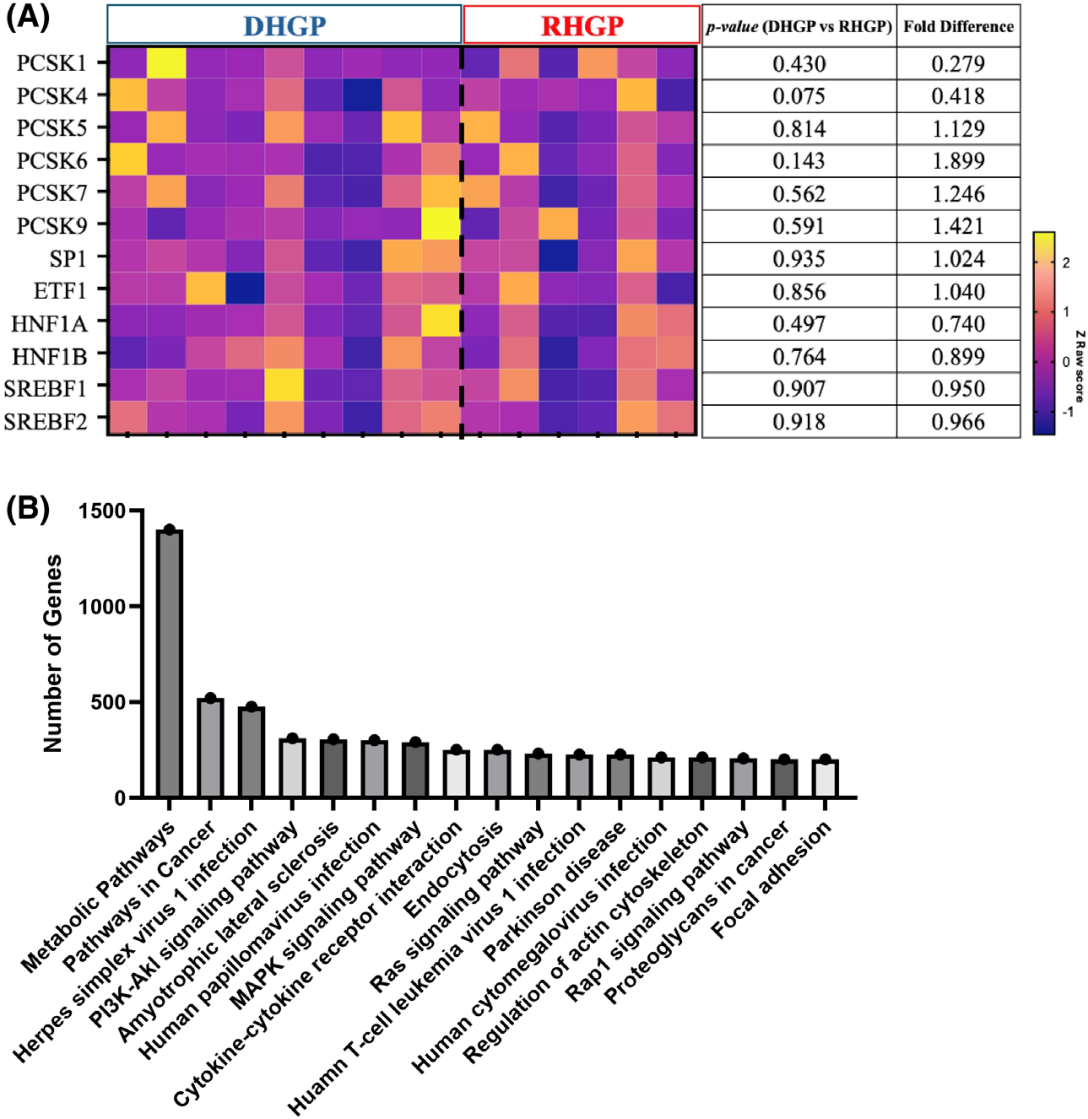
Expression of PCSK genes and genes involved in cholesterol and lipid regulation in RHGP and DHGP colorectal cancer liver metastasis. (A) Heatmap displaying *Z*‐score–normalized expression of Proprotein Convertase Subtilisin/Kexin (PCSK) family genes and key genes involved in cholesterol and lipid regulation in colorectal cancer liver metastasis (CRCLM) with desmoplastic histological growth pattern (DHGP; *n* = 9) or replacement histological growth pattern (RHGP; *n* = 6) histologic growth patterns. Rows represent genes and columns represent individual tumor samples, with red indicating a higher expression and green indicating a lower expression relative to the mean. Unpaired *t*‐test, corresponding *P*‐values and fold differences (DHGP vs. RHGP) are listed in the adjacent table. (B) Pathway abundance analysis bar plot showing the number of genes associated with each annotated biological pathway across all samples when comparing RHGP versus DHGP. Pathways were ranked by total gene count derived from Gene Set Enrichment Analysis (GSEA).

Building upon the observed differences in serum cholesterol, we conducted a deeper analysis looking into the gene expression profiles of CRCLM patients, where we focused on genes known for their involvement in cholesterol and lipid regulation, particularly the Proprotein Convertase Subtilisin/Kexin family (e.g., PCSK9 and PCSK7), as well as SP1, E2F1, HNF1A, HNF1B, SREBF1, and SREBF2. Despite their well‐documented roles in cholesterol metabolism, no significant differences in the expression of these genes were observed between replacement and desmoplastic HGPs groups, reflecting the lack of distinction found in serum cholesterol and LDL profile (Fig. 2A).

However, pathway‐level analysis revealed notable differences in metabolic processes across the HGPs. Among the most prominently enriched pathways were those related to general metabolism, as shown in Fig. 2B, which includes gene networks involved in lipid biosynthesis, fatty acid oxidation, and energy homeostasis. Although individual lipid‐related genes such as *PCSK9* and *PCSK7* did not exhibit significant differential expression, the enrichment of metabolic pathways suggests broader regulatory shifts in lipid metabolism that may distinguish the HGPs. These findings could influence tumor progression and microenvironment interactions depending on the predominant growth pattern.

### Statin administration attenuates the development of vessel co‐opting liver metastatic tumors

**Fig. 3:**
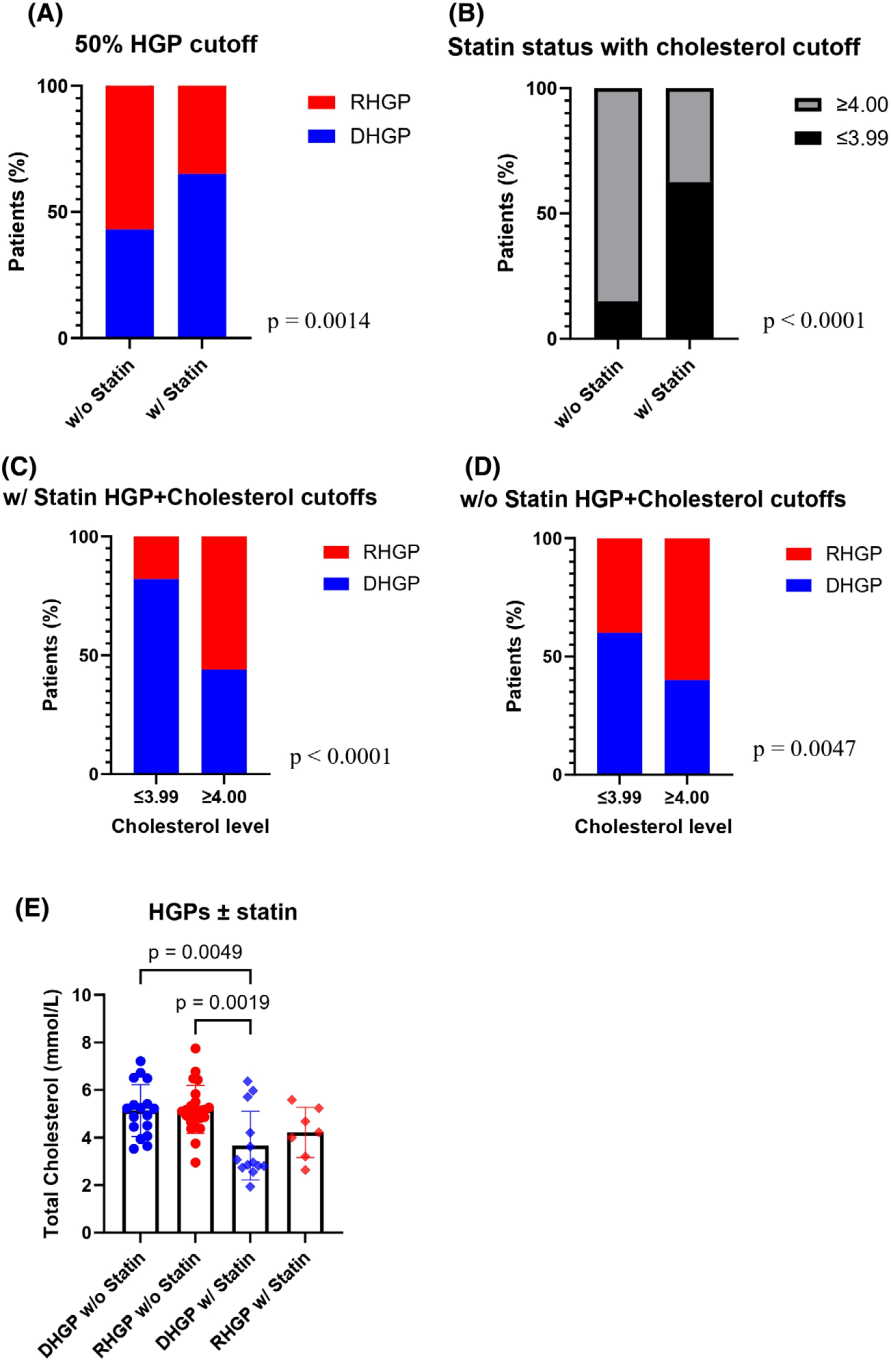
Statin effect on HGPs dispersion and total serum cholesterol. (A) Patients' percentage stratification by statin intake, each bar represents the percentage of colorectal cancer liver metastasis (CRCLM) patients with different types of histological growth patterns (HGPs). (*n* = 60). (B) Patients' percentage stratification by statin intake, each bar represents the percentage of CRCLM patients with cholesterol stratification of 3.99 mmol·L^−1^ and lower, or 4.00 mmol·L^−1^ and higher. Chi‐squared test, *P* < 0.0001. (C, D) Statin treated (B) (*n* = 20) and untreated (C) (*n* = 40) patients' percentage stratification by cholesterol levels, each bar represents the percentage of CRCLM patients with different types of HGPs. HGPs cutoffs used for these figures are at 50%. Chi‐square test for A–D, (A) *P* = 0.0014, (B) *P* < 0.0001, (C) *P* < 0.0001, (D) *P* = 0.0047. (E) Total serum cholesterol of patients with and without statin intake, with desmoplastic histological growth pattern (DHGP) or replacement histological growth pattern (RHGP). One‐way ANOVA (*n* = 60). Error bars denote standard deviation.

The primary source of circulating cholesterol is the liver, and HMGCR serves as a key regulatory enzyme controlling cholesterol synthesis [42]. 3‐Hydroxy‐3‐methylglutaryl coenzyme A reductase (HMGCR) is the rate‐limiting enzyme in the *de novo* synthesis of cholesterol and serves as a key regulatory enzyme controlling endogenous cholesterol synthesis. It is a known therapeutic target of statins and has been used clinically to treat hypercholesterolemic patients [43]. We observed that some patients in our cohort also used statins to reduce their high cholesterol levels. Thus, we decided to categorize the patients into two groups including patients not taking statins (*n* = 40) and those who use statins (*n* = 20) and investigate whether there was a correlation with HGPs, focusing on the two main HGPs at a 50% cutoff. Interestingly, we demonstrated a significant reduction in the proportion of patients with RHGP lesions in the group of patients using statins (Fig. 3A). To further examine the association between RHGP and statin administration in CRCLM patients, we compared the levels of total cholesterol in the patients treated with statins to the rest of the patients.

The administration of statins significantly lowered total cholesterol in the serum of patients (Fig. 3B), which interestingly corresponded with an increase in DHGP (*n* = 13) vs RHGP (*n* = 7), in those patients treated with statins (Fig. 3C). In untreated patients, presenting higher cholesterol levels, we observed an increase in RHGP (*n* = 23) vs DHGP (*n* = 17) (Fig. 3D). No significant difference was observed between RHGP patients treated and untreated with statin, as well as between HGPs of treated or untreated samples (Fig. 3E), when we did not use the 4 mmol·L^−1^ cutoff. Thus, the levels of cholesterol are critical for this significant finding.

### Inhibition of PCSK9 via Evolocumab reduces the establishment of vessel co‐option tumors

**Fig. 4:**
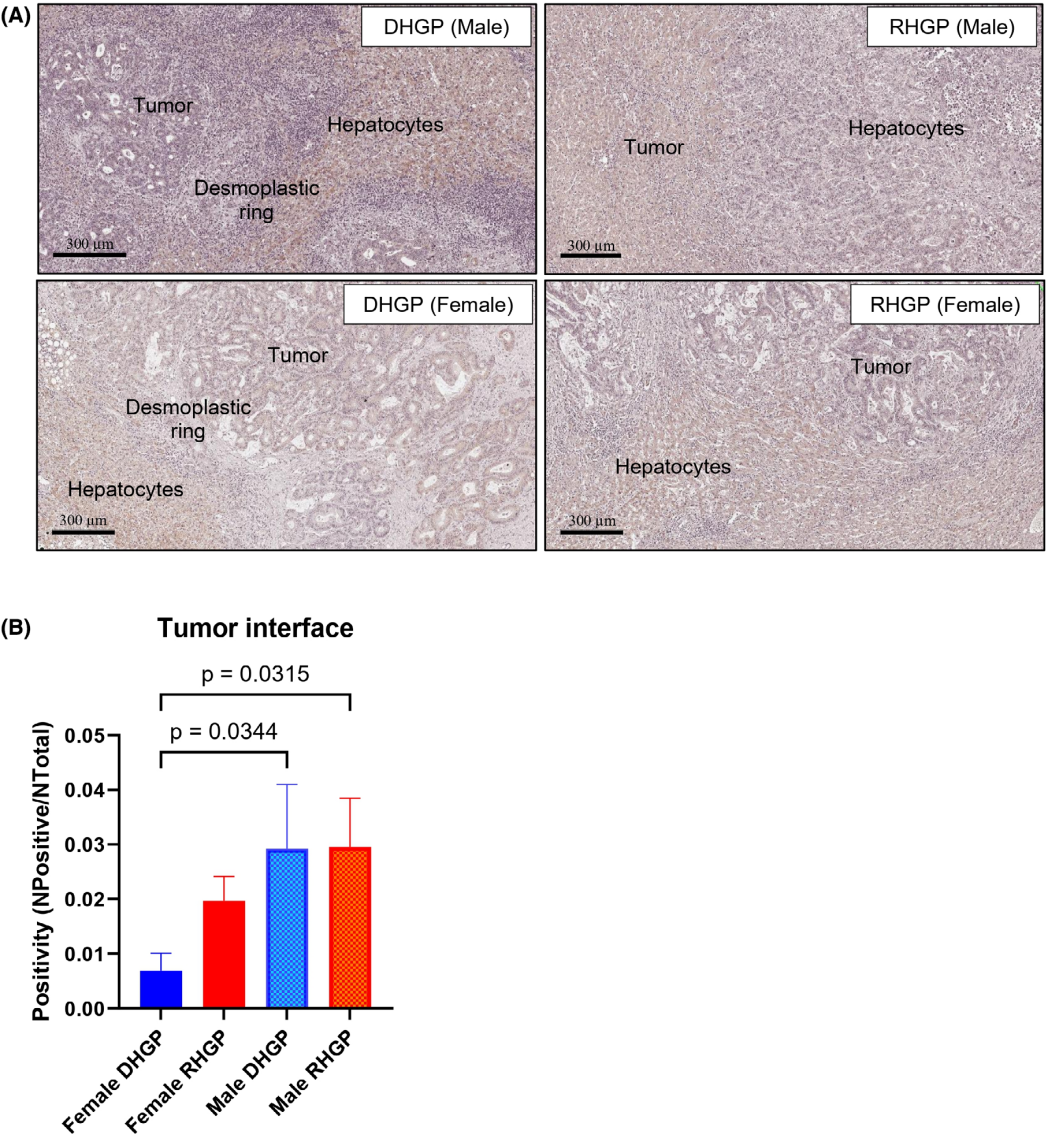
Immunohistochemical staining of chemo‐naïve colorectal cancer liver metastasis (CRCLM) patients using anti‐hPCSK9 antibody9. (A) Representative immunohistochemical images of desmoplastic histological growth pattern (DHGP) and replacement histological growth pattern (RHGP) in female and male samples. (B) Positivity results of antihuman proprotein convertase subtilisin/kexin type 9 (anti‐hPCSK9) presenting difference of PCSK9 presence at the tumor‐liver interface. One‐way ANOVA (*n* = 60). Error bars denote standard deviation.

PCSK9 and LDLR (low‐density lipoprotein receptor) are key players in cholesterol metabolism, specifically in the regulation of low‐density lipoprotein (LDL) cholesterol levels in the blood. PCSK9 promotes the degradation of LDLR, leading to increased levels of LDL cholesterol, while inhibiting PCSK9 can lower LDL cholesterol. Thus, another mechanism involved in hyperlipidemia is the overexpression of PCSK9, which degrades LDLR and reduces the absorption of LDL by the liver [10, 39, 44, 45]. We performed immunohistochemical staining of PCSK9 on chemo‐naïve patients with RHGP (*n* = 11) and DHGP (*n* = 9) (Fig. 4A), to investigate a possible mechanism linked to the blood cholesterol differences we observed. Our immunohistochemical analysis showed no significant difference when comparing DHGP to RHGP lesions; however, we did observe significant differences once we stratified the samples based on sex. The PCSK9 levels in DHGP lesions from female patients were significantly lower in the tumor tissue compared to RHGP lesions in females and lower when compared to either DHGP or RHGP lesions in males (Fig. 4B). Interestingly, this does not correlate with the serum cholesterol, as low levels of PCSK9 in tissue would suggest less cholesterol is available in circulation, which does not correlate with what we show that females have higher levels of serum cholesterol. This could suggest that tumor PCSK9 levels are not predictive or correlate with circulating cholesterol.

**Fig. 5:**
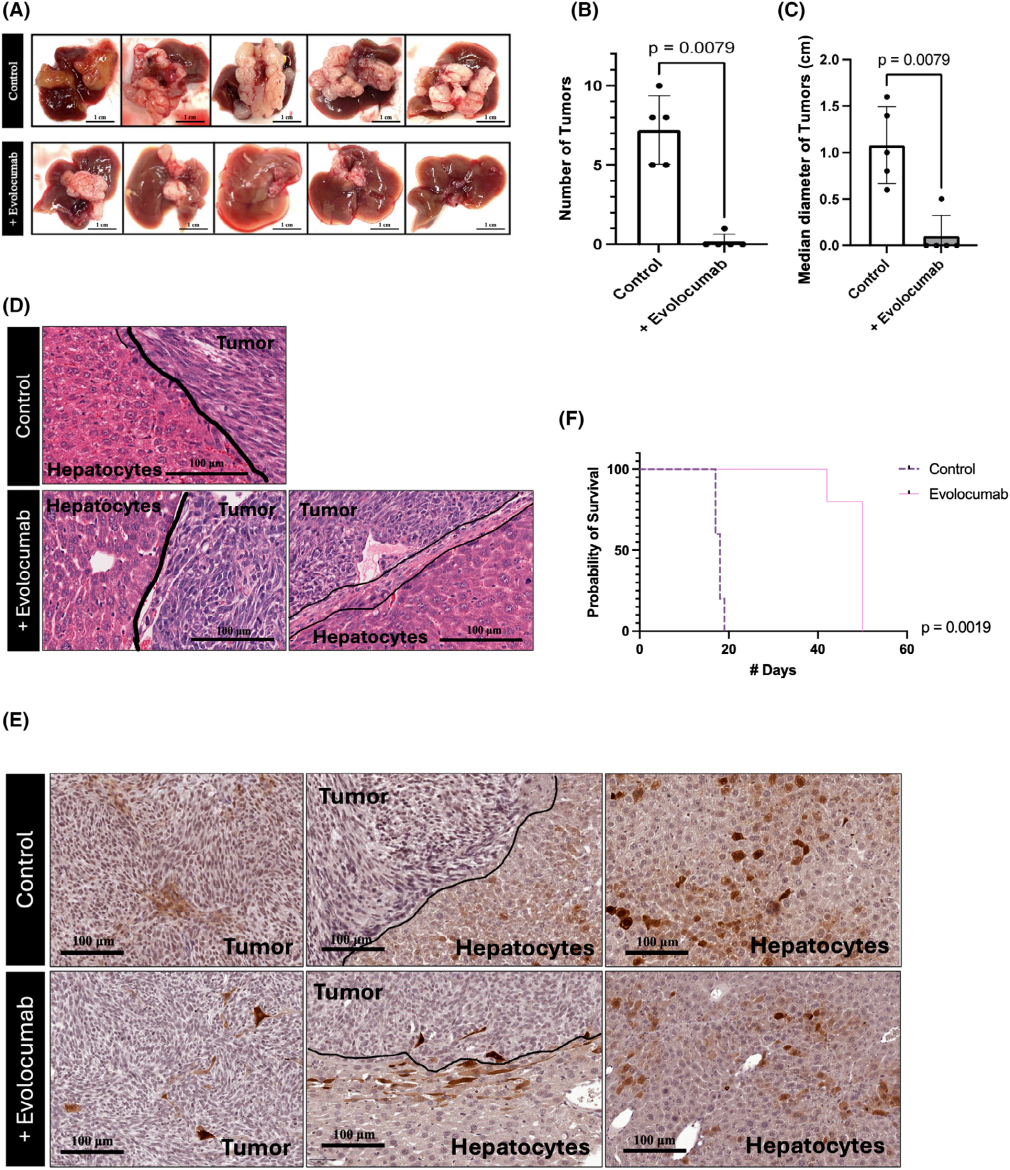
Inhibition of PCSK9 through evolocumab stimulates the development of angiogenic CRCLM tumors *in vivo*. (A) Images of the liver of control (untreated mice) and treated mice with anti‐Proprotein Convertase Subtilisin/Kexin type 9 (anti‐PCSK9) (evolocumab) that received intrasplenic injections of MC38 cells. Scale bar of 1 cm. (B, C) Represent the number and diameter of the generated liver metastatic tumors, respectively. Unpaired *t*‐test, *P* = 0.0079, *P* = 0.0079 (*n* = 10). (D) Hematoxylin and eosin (H&E) staining of the generated lesions. Scale bar of 100 μm. (E) Immunohistochemical staining of MC38 colorectal cancer liver metastasis (CRCLM) mouse model sections using an anti‐PCSK9 antibody. Scale bar of 100 μm. (F) Kaplan–Meier survival curves comparing the two groups. Log‐rank test, *P* = 0.0019 (*n* = 10). Error bars denote standard deviation. The black lines in D and E delineate the tumor to hepatocyte interface. Double black line delineates the desmoplastic ring.

Since PCSK9 shows a trend of higher levels in female patients with RHGP lesions compared to female patients with DHGP, we questioned whether PCSK9 may influence the development of RHGP tumors. To address this, we used a liver metastatic animal model [46, 47]. C57BL/6 mice with liver metastases were treated with evolocumab, an anti‐PCSK9 monoclonal antibody that is approved to treat hyperlipidemia in human patients. After intrasplenic injection with colorectal carcinoma MC38 cells, the mice were injected with either evolocumab (200 μg per every 2 days) or vehicle for two weeks. Significantly, treatment with evolocumab resulted in a reduction in the generation of lesions (Fig. 5A). Whereas all control mice developed multiple lesions in their liver, only one evolocumab‐treated mouse out of five developed a single lesion (Fig. 5A–C). Fig. 5B depicts the analysis, based on the number of lesions, demonstrating a significant reduction, with four out of five mice having a complete response. Interestingly, while the control mice developed RHGP lesions, the single mouse in the treated group that developed a lesion, demonstrated a mixed lesion with a portion having a desmoplastic ring, similar to DHGP lesions in humans. Thus, treatment with evolocumab has converted the infiltrating RHGP lesion into a DHGP as demonstrated by a ring around the lesion (Fig. 5D). We also performed immunohistochemistry using an anti‐PCSK9 antibody. In the control group, PCSK9 levels are generally lower in tumor tissue compared to hepatocytes, with both central and peripheral tumor regions showing significantly reduced levels compared to peripheral normal tissue. Similarly, in samples treated with evolocumab, PCSK9 levels in tumors remain lower than those in hepatocytes. Notably, only one out of five mice developed a tumor following evolocumab treatment. Due to the presence of only a single lesion across all mice, statistical analysis could not be performed on the expression levels of PCSK9 as they would represent the single lesion only (Fig. 5E). However, there was an observable trend indicating that the PCSK9 levels in the lesion of the evolocumab‐treated mouse were lower than those in the control group. We also plotted the survival curves of these mice (Fig. 5F) and while the control mice died within 28 days, the treated mice survived up to more than 50 days at which time they were euthanized, even though they showed no signs of distress.

## Discussion

**Fig. 6:**
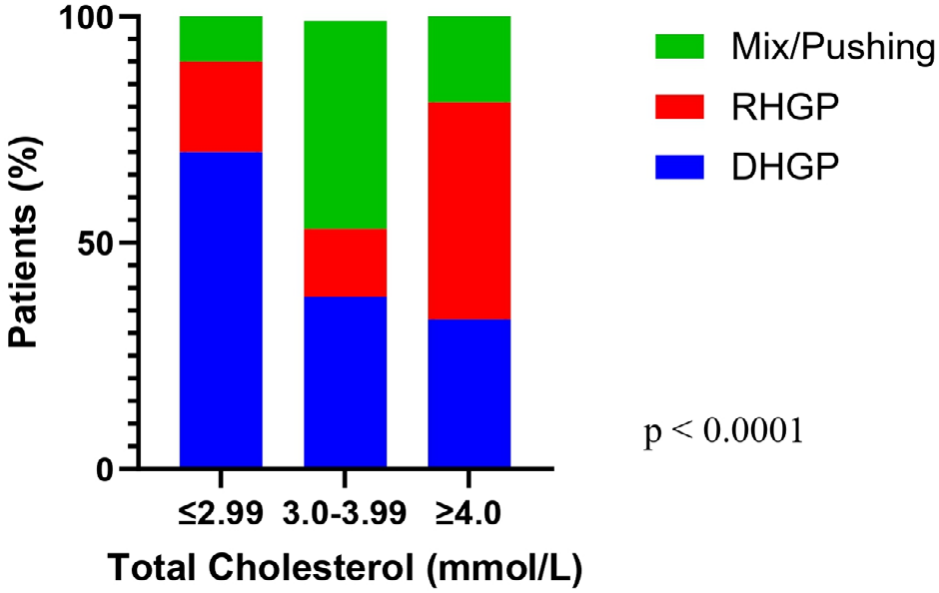
Stratification of HGPs in colorectal cancer liver metastasis (CRCLM) patients by Serum Cholesterol levels. Patients' percentage stratification by 50% cutoff histological growth patterns (HGPs) divided based on the concentration of total serum cholesterol. Chi‐squared test, *P* < 0.0001 (*n* = 77).

Elevated serum levels of total cholesterol and LDL have been reported to have a negative prognostic impact in various cancers, such as breast [48], colon, gastric, and ovarian [49]. Our clinical data demonstrated that high levels of serum total cholesterol and LDL are associated with the RHGP CRCLM. Additionally, our data suggest that PCSK9 is a key molecule in the development of RHGP lesions, and its inhibition attenuated the development of RHGP lesions *in vivo*. To our knowledge, this is the first report to show the function of PCSK9 in the development of liver metastases, specifically in CRCLM. These data have significant translational potential. First, our data suggest the possibility of using serum cholesterol data profiles to predict the presence of RHGP tumors, which are driven by co‐option and have an overall poor prognosis. In addition, our results suggest pushing [38] as a transition HGP between DHGP and RHGP when cholesterol ranges between 3.00 and 3.99 (Fig. 6). Second, our results demonstrate that targeting circulating PCSK9 is a compelling rationale for conducting future clinical trials in CRCLM patients, particularly those patients that have RHGPs, which are resistant to antiangiogenic therapy and have poor 5‐year overall survival with current standard of care.

Interestingly, Pelton *et al*. [50] have suggested that hypercholesterolemia promotes tumor angiogenesis in breast cancer. Similar results were proposed in hepatocellular carcinoma [51] and prostate cancer [52]. In contrast, our results suggested that hypercholesterolemia impairs the development of DHGP lesions, which are primarily angiogenic tumors [53]. Those patients with high cholesterol had RHGP lesions, which we have previously published, that obtain their blood supply by hijacking existing blood vessels (vessel co‐option). It is worth mentioning that hypercholesterolemia has been shown to inhibit angiogenesis via the inhibition of endothelium‐derived nitric oxide (EDNO) release [44], which is known as a mediator of angiogenesis [54]. Our preclinical data showed that treatment with anti‐PCSK9, known to treat high cholesterol, prevents the growth of non‐angiogenic lesions (RHGP) and may promote the growth of angiogenic lesions, as we observed that the only lesion growing in the mouse developed a desmoplastic ring, similar to DHGP in human CRLCM, which are angiogenically driven. The function of PCSK9 in tumor angiogenesis is poorly understood. Abdelwahed *et al*. [55, 56] have reported that inhibition of PCSK9 suppressed tumor angiogenesis in prostate cancer. Interestingly, another study has linked PCSK9 overexpression to resistance against sorafenib, an antiangiogenic agent, in HCC [57]. However, this study has not examined the mechanistic role of PCSK9 in vessel co‐option (i.e., RHGP lesions). Herein, we suggested a positive correlation between PCSK9 upregulation and vessel co‐option (RHGP) in CRCLM. However, further studies are required to identify the role of PCSK9 in the development of vessel co‐option and the *in vivo* experiments need to be performed in female mice as it has been shown that in mice, opposite to humans, males have higher levels of cholesterol than females. This may affect the response to PCSK9 inhibitors.

## Materials and methods

### Clinical data and patient samples

The study was conducted in accordance with the guidelines approved by McGill University Health Centre Institutional Review Board (IRB), ethics protocol MUHC‐11‐066‐SDR, and conforms with the Declaration of Helsinki. Informed and written consent was obtained from all patients through the McGill University Health Centre (MUHC) Liver Disease Biobank. This study was performed on 116 CRCLM patients who were referred to McGill University Health Centre (MUHC) in Montreal. Blood samples used in this study were collected between June 2013 and February 2021. For patient characteristics, see Table 1. HGPs characterization was successfully assessed in 107 patients [58]. For the HGP cutoff, as most patients have between 50% and 70% of either HGP, we decided to use a 50% cutoff to have a sufficient number of patients for statistical power. When performing the analysis with a 100% cutoff, we observed the same results; nevertheless, we had fewer samples per group. Therefore, to include all patients in this study we used a 50% cutoff. Furthermore, we and others have shown that a 50% cutoff correlates with overall survival and therefore is a more appropriate cutoff for these studies [36].

**Table 1:** Demographic baseline of CRCLM patients.

	Characteristic	Value	Percentage (%)
1	**Sex**		
	Male	74	63.79
	Female	42	36.21
2	**Age**		
	< 50 years	3	2.59
	50–70 years	63	54.31
	> 70 years	50	43.10
3	**Body mass index category (BMI)**		
	Underweight	0	0.00
	Normal	48	41.38
	Overweight	39	33.62
	Obese	27	23.28
	Insufficient data	2	1.72
4	**Histopathological growth pattern (HGP) (50% predominant HGP cutoff)**		
	Replacement HGP (RHGP)	42	39.25
	Desmoplastic HGP (DHGP)	40	37.38
	Mixed	25	23.36
5	**Neoadjuvant before liver resection**		
	Yes	96	89.72
	No	18	16.82
	Insufficient data	2	1.87
6	**Liver metastasis recurrence**		
	Yes	49	42.24
	No	67	57.76

Cholesterol levels were measured in 87 patients, of whom 77 overlapped with the HGP patients. Surgical specimens were procured and released to the Biobank immediately after the pathologist's confirmation of carcinoma and surgical margins. Those patients receiving cholesterol‐lowering medications included the following statin treatments: atorvastatin and rosuvastatin.

### RNA sequencing

RNA sequencing data are documented in Palmieri and Metrakos *et al*. [37]. Briefly, total RNA was extracted from 15 chemotherapy‐naive colorectal cancer liver metastasis (CRCLM) lesions and paired normal liver tissues. After sample clustering analysis, RNA quality and quantity were assessed, and libraries were prepared for whole transcriptome sequencing using Illumina TruSeq RNA kits. For each sample, 2 μg of RNA was reverse transcribed with poly (dT) primers and SuperScript II. Library quality and quantity were measured using a Bioanalyzer and Qubit, followed by 100‐bp paired‐end sequencing on the HiSeq 2500 platform (Illumina). For visualization, normalized gene expression values were *Z*‐score standardized by gene across all samples and plotted as a heatmap. The heatmaps were generated using graphpad prism software version 10.0 (GraphPad Software, La Jolla, CA, USA). Gene Set Enrichment Analysis (GSEA) was performed using the GSEA software to identify significantly enriched biological pathways and gene sets associated with replacement and desmoplastic HGPs.

### Immunohistochemistry staining

We performed immunohistochemical staining for formalin‐fixed paraffin‐embedded (FFPE) specimens as described in previous publications [53, 59]. Briefly, resected CRCLM patient samples and mice liver samples were formalin‐fixed and paraffin‐embedded (FFPE), then sectioned at 4 μm for histopathological evaluation. Following fixation in paraformaldehyde and embedding, sections were placed on charged slides, with the first section H&E stained. Deparaffinization and rehydration preceded antigen retrieval, peroxidase blocking, and serum blocking. Overnight incubation with primary antibody staining was performed using in‐house polyclonal PCSK9 antibody 1 : 1500 (generous gift from Dr Nabil G Seideh), followed by the EnVision+ System‐HRP kit (Dako, K4007; Agilent Technologies, Mississauga, ON, Canada) detection system. Sections were counterstained with hematoxylin prior to dehydration and mounted with Permount (Fisher, SP‐15‐100; Saint‐Laurent, QC, Canada).

### Immunohistochemistry quantification

To quantify the immunohistochemistry staining, we used the Aperio ImageScope version 11.2.0.780 software program for scoring analysis. The Aperio V9 Algorithm was used to measure the intensity of the staining. The positivity [Total number of positive pixels divided by the total number of pixels: (NTotal – Nn)/(NTotal)] was assessed for three regions of interest (ROI) for central tumor, peripheral tumor, peripheral normal and distal normal, and then the average positivity was determined for each group.

### Syngeneic mouse experiments

CRCLM tumors were generated in male C57B/6 mice, aged between 6 and 7 weeks, using an intrasplenic injection protocol as described in previous publications [46, 47]. The animals were obtained from the inbred colony maintained by the Animal Resources Division (ARD) of the Research Institute of the McGill University Health Centre (RI‐MUHC). The ARD ensures full compliance with the standards of the Canadian Council on Animal Care (CCAC). Animals were housed in a pathogen‐free facility under a controlled 12‐h light/dark cycle and were grouped in 4 per cage. Each cage was autoclaved prior to use and equipped with sterilized bedding and a continuous supply of water. All experimental procedures involving animals were performed in accordance with the CCAC guidelines and approved under protocol MUHC‐7841.

The mice were divided into two groups 5 days after the intrasplenic injection protocol using MC38 cells. The mice in the first group were injected with a vehicle alone (Control). The mice in the second group were treated with 200 μg of Evolocumab (Repatha; Amgen Manufacturing Limited) every two days. Evolocumab was a generous gift from Dr Nabil G. Seideh. It is important to note that this model only develops replacement‐type HGP lesions.

### Statistical reproducibility

Statistical analysis was performed using the graphpad prism software version 10.0 (GraphPad Software, La Jolla, CA, USA). One‐way ANOVA, followed by Tukey's multiple comparison tests, was used to compare the means of several groups. Unpaired Student's *t*‐test was applied to compare the means of two groups. The association between the two categorical groups was assessed with the chi‐squared test. Overall survival estimates for the *in vivo* experiments were calculated using the Kaplan–Meier model from the date of inoculation to the date of death. The significance was calculated using the Log‐Rank test. *P*‐values of < 0.05 were considered significant. Data presented as mean ± standard deviation.

## Author contributions

MR, MT, AL, NGS and PM co‐conceived the study. LK and SP collected the clinical data. SP prepared CRCLM samples. MR and AKL performed the cell culturing, xenograft experiments and mice experiments. MR, AG, AT, and AK‐L performed immunohistochemistry, immunofluorescence, and data curation. AG, AK‐L, and AL prepared and wrote the manuscript. AL, NGS, and PM reviewed the manuscript.

## Conflict of interest

The authors declare no conflict of interest.

## Data Availability

All data produced in the present study is available upon reasonable request to the authors.
